# Clear Shot at Better Outcomes?: Closure of Coal-Burning Plants Could Improve Neurodevelopment

**Published:** 2008-10

**Authors:** Tanya Tillett

Coal burning, which provides up to 75% of China’s electricity, is the main environmental source of polycyclic aromatic hydrocarbons (PAHs) in that country. Research in Europe, the United States, and Asia indicates that prenatal exposure to PAHs increases the risk of reduced fetal growth and adverse neurodevelopmental effects. Cofirmation for these studies comes in new research in China, which suggests that reduction of prenatal exposure to PAHs was linked to improved developmental outcomes in a small group of Chinese children **[*EHP* 116:1396–1400; Perera et al.]**.

The study took advantage of the opportunity to evaluate health-related effects of the prescheduled closure of a coal-fired power plant. Subjects included about 110 children in each of two parallel mother–infant cohorts in Tongliang, Chongqing Province. The first (2002) cohort was enrolled two years before the 2004 shutdown of the power plant; the second (2005) cohort was enrolled the year after the shutdown.

The infants were followed from birth through their second birthdays, at which time the investigators assessed the children’s developmental attainment using the Gesell Developmental Schedules. Using high-performance liquid chromatography, they analyzed PAH–DNA adduct levels in cord blood collected at delivery and also measured potential confounders for neurodevelopmental effects, including lead, mercury, and secondhand tobacco smoke. The relationships between PAH–DNA adduct levels and developmental outcomes in the two cohorts were evaluated through the use of multiple linear regression and logistic regression, adjusting for potential confounders. Cohort developmental outcomes, including frequency of developmental delay, also were compared.

The investigators found that the 2005 cohort had 40% lower PAH–DNA adduct levels in cord blood compared with the 2002 cohort. Earlier studies of the 2002 cohort showed significant associations between elevated adduct levels and lower average and motor development scores; however, these associations were not observed in the 2005 cohort. The frequency of developmental delay in the motor area was significantly reduced in the 2005 cohort compared with the 2002 cohort.

The results are limited by the lack of data on postnatal PAH–DNA adduct levels. However, the authors note that in 2002 the plant was the major source of PAH emissions in the study area (residential heating and cooking had been converted to natural gas). Thus, the results provide molecular epidemiologic evidence that developmental outcomes in infants were improved after the coal-burning power plant was shut down—a finding relevant to child development throughout China as well as other countries relying on coal and other fossil fuels for energy.

## Figures and Tables

**Figure:**
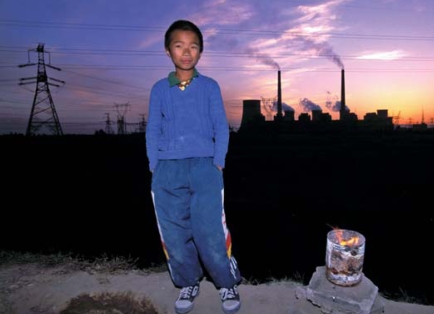
Coal-fired power plants such as the Datong facility in the background provide 75% of China’s electricity.

